# Assessing information provided via artificial intelligence regarding distal biceps tendon repair surgery

**DOI:** 10.1002/jeo2.70281

**Published:** 2025-05-19

**Authors:** Suhasini Gupta, Brett D. Haislup, Ryan A. Hoffman, Anand M. Murthi

**Affiliations:** ^1^ T.H. Chan School of Medicine University of Massachusetts Worcester Massachusetts USA; ^2^ Department of Shoulder and Elbow Surgery MedStar Union Memorial Hospital Baltimore Maryland USA

**Keywords:** artificial intelligence, distal biceps repair, patient education, technology in orthopaedic procedures

## Abstract

**Purpose:**

The purpose of this study was to analyze the quality, accuracy, reliability and readability of information provided by an Artificial Intelligence (AI) model ChatGPT (Open AI, San Francisco) regarding Distal Biceps Tendon repair surgery.

**Methods:**

ChatGPT 3.5 was used to answer 27 commonly asked questions regarding ‘distal biceps repair surgery’ by patients. These questions were categorized using the Rothwell criteria of *Fact, Policy* and *Value*. The answers generated by ChatGPT were analyzed using the DISCERN scale, *Journal of the American Medical Association* (JAMA) benchmark criteria, Flesch‐Kincaid Reading Ease Score (FRES) and grade Level (FKGL).

**Results:**

The DISCERN score for *Fact*‐based questions was 59, *Policy* was 61 and *Value* was 59 (all considered ‘good scores’). The JAMA benchmark criteria were 0, representing the lowest score, for all three categories of *Fact, Policy* and *Value*. The FRES score for the *Fact* questions was 24.49, *Policy* was 22.82, *Value* was 21.77 and the FKGL score for *Fact* was 14.96, *Policy* was 14.78 and *Value* was 15.00.

**Conclusion:**

The answers provided by ChatGPT were a ‘good’ source in terms of quality assessment, compared to other online resources that do not have citations as an option. The accuracy and reliability of these answers were shown to be low, with nearly a college‐graduate level of readability. This indicates that physicians should caution patients when searching ChatGPT for information regarding distal biceps repairs. ChatGPT serves as a promising source for patients to learn about their procedure, although its reliability and readability are disadvantages for the average patient when utilizing the software.

**Abbreviations**
AI: artificial intelligence
FKGL: Flesch‐Kincaid Grade Level
FRES: Flesch‐Kincaid Reading Ease Score
JAMA: Journal of the American Medical Association
NIH: National Institutes of Health

## INTRODUCTION

Distal biceps tendon ruptures are common upper‐extremity injuries, with a national incidence of 2.5 per 100,000 patient‐years, or a rate of 0.0025% distal biceps tendon ruptures in the United States [10]. A distal biceps tendon rupture is an injury that occurs when excessive eccentric tension is applied to a flexed elbow and can occur with activities such as waterskiing, golf, wrestling, basketball or lifting heavy weights or objects [10]. Men account for the vast majority of distal biceps ruptures (95%), especially between the ages of 35 and 54 years [10]. For most patients, surgery is indicated as it can help improve the deficits in supination and flexion caused by the tendon rupture. But some patients who are not as physically active, or have other co‐morbidities, may choose a non‐operative approach [10, 17]. As such, patients typically need guidance in both education regarding their diagnosis, as well as next steps for treatment [17].

Orthopaedic literature has shown that patients turn to the internet, perioperatively and often use websites without peer review or other fact‐checking methods to obtain information regarding surgery [9, 13]. It has been shown that patients who search online sources for medical information think of this information as equivalent to or even better than the information provided by their physician. Patients use these online sources of information to learn about time of recovery, degree of function after surgery, technical details of the surgery and activity restrictions post‐operatively [18]. Additionally, a large percentage of patients do not corroborate research information with their physician [4]. Such studies have found the information available on these platforms to be of various accuracy, reliability and readability [3, 6, 13, 18, 20].

Technological models, such as ChatGPT (OpenAI, San Francisco) use machine learning and supervised deep learning to provide patients with medical information [1, 3, 8, 12, 19, 23]. There have been multiple studies within the orthopaedic literature that have shown that information collected from ChatGPT can be useful but must be used with caution due to concerns about reliability and readability [3, 21]. With the increasing availability of artificial intelligence (AI) sources such as ChatGPT, it is essential to continue to evaluate the quality of the information that ChatGPT provides to patients.

The purpose of this investigation was to analyze the quality, accuracy and readability of information provided by ChatGPT regarding commonly asked questions by patients regarding distal biceps tendon repairs. We hypothesize that ChatGPT will be able to provide adequate supplementary information regarding distal biceps tendon repair but will be unable to do so at the appropriate reading levels for patients.

## METHODS

Twenty‐seven commonly asked questions, related to distal biceps tendon ruptures, were collected from various studies or academic websites, and modified to fit distal biceps tendon repairs [7, 11, 18]. The phrasing ‘distal biceps repair surgery’ was used to input questions into ChatGPT 3.5 to mimic verbiage used by patients. These questions were sorted based on the Rothwell Criteria, within categories of *Fact, Policy* and *Value*. A *Fact*‐based question asks whether something is true or not. *Policy*‐based question asks whether a specific course of action can be used to solve a certain problem. *Value‐*based questions ask for an evaluation of an idea, object or event [14]. The questions inputted into ChatGPT (OpenAI, San Francisco) can be found in Table 1, and their respective answers are available in Appendix S1. The answers were then assessed for quality via the DISCERN scale, accuracy via the *Journal of the American Medical Association* (JAMA) benchmark criteria, and readability via the Flesch‐Kincaid Reading Ease Score (FRES) and Flesch‐Kincaid Grade Level (FKGL).

**Table 1:** Commonly asked questions regarding distal biceps tendon repair, categorized by Rothwell criteria.

Rothwell criteria	Distal biceps tendon repair (distal biceps repair surgery)
Fact	1. How do you go to the bathroom after distal biceps repair surgery? 2. Can you play sports after distal biceps repair surgery? 3. What is the success rate of distal biceps repair surgery? 4. What are the most common complications of a distal biceps repair surgery? 5. What can you not do after distal biceps repair surgery? 6. When can I begin driving after a distal biceps repair surgery? 7. Can I take my splint off to sleep after distal biceps repair surgery? 8. Can I have an MRI after distal biceps repair surgery? 9. Where is the incision for distal biceps repair surgery? 10. How long should you take off work after distal biceps repair surgery? 11. How long does it take to recover from distal biceps repair surgery? 12. How long is the physical therapy after distal biceps repair surgery? 13. What is the average age for distal biceps repair surgery? 14. Can you throw a ball after distal biceps repair surgery? 15. How much does Medicare pay for distal biceps repair surgery?
Policy	16. Can you wait too long for distal biceps repair surgery? 17. Are there alternatives to distal biceps repair surgery? 18. What is the fastest way to recover from distal biceps repair surgery? 19. How can I avoid distal biceps repair surgery? 20. Is distal biceps repair surgery an inpatient or outpatient procedure? 21. What is the chance of dying in a distal biceps repair surgery?
Value	22. Why is distal biceps repair surgery so painful? 23. What happens if you don't wear your splint after a distal biceps repair surgery? 24. Can you ice too much after a distal biceps repair surgery? 25. How painful is a distal biceps repair surgery? 26. How long does a distal biceps repair surgery? 27. Are distal biceps repair surgery successful?

The DISCERN scale, which was developed by the British Library, is a quality assessment tool used by several similar studies to assess the quality of information provided by a source related to health education [2, 8, 21]. The DISCERN scale is divided into three sections, and 16 questions which assess the reliability, quality of information provided on treatment choices and overall rating of the publication. Each of the 16 questions is graded on a scale of 1–5, with 1 being the lowest score indicating poorest quality or ‘no’ and 5 indicating the highest quality or ‘yes’. The summation of the answers yields a score out of 80, wherein a score of 50 is considered ‘good’ and a score over 70 is considered ‘excellent’ [2]. Two authors graded all the answers provided by ChatGPT on the DISCERN scale (Table 2). Two authors, SG and BH, independently scored the questions, and discussed any discrepancies.

**Table 2:** DISCERN scores for questions, categorized by Rothwell criteria.

DISCERN scale	Fact	Policy	Value
**Section 1: Is this publication reliable**			
Are the aims clear?	3	3	3
Does it achieve its aims?	4	4	4
Is it relevant?	5	5	5
Is it clear what sources of information were used to compile the publication (other than the author or producer)	1	1	1
Is it clear when the information used or reported in the publication was produced?	1	1	1
Is it balanced and unbiased?	3	3	3
Does it provide details of additional sources of support and information?	1	1	1
Does it refer to areas of uncertainty?	5	5	5
**Section 2: How good is the quality of information on treatment choices?**			
Does it describe how each treatment works?	4	4	4
Does it describe the benefits of each treatment?	5	4	5
Does it describe the risks of each treatment?	5	5	5
Does it describe what would happen if no treatment is used?	3	5	3
Does it describe how the treatment choices affect overall quality of life?	5	5	5
Is it clear that there may be more than one possible treatment choice?	4	5	4
Does it provide support for shared decision‐making?	5	5	5
**Section 3: Overall rating of the publication**			
Based on the answers to all the above questions, rate the overall quality of the publication as a source of information about treatment choices	5	5	5
**Total**	59	61	59

The JAMA benchmark criteria are a tool used to assess the reliability of a publication, also widely used in similar studies [8, 16]. The score comprises of four components; Authorship which identifies the person or group that produces the information, Attribution which assesses if citations and references are provided, Disclosures which assesses if information regarding who ‘owns’ the information is available, and Currency assesses if dates for when the information was last reviewed or posted are provided [16, 24].

Finally, the FRES and FKGL are used to assess readability and are used for the same in the American education system [21]. The FRES score is out of 100, with lower numbers indicating a more complex text, and is calculated by the formula 206.835 − 1.015 × (total words/total sentences) − 84.6 × (total syllables/total words). The FKGL score, which is an adaptation of the FRES score, measures the minimum grade level required by the reader, and is calculated by the score 0.39 × (total words/total sentences) + 11.8 × (total syllables/total words) − 15.59. A higher FKGL number indicates a higher minimum grade level required to read the text.

## RESULTS

### Quality assessment

The DISCERN score for *Fact*‐based questions was 59, *Policy* was 61 and *Value* was 59. Since these scores are all over 50, they are considered as ‘good’ sources of information.

### Accuracy assessment

The *JAMA benchmark criteria* were 0 for *Fact, Policy and Value* questions as the answers provided by ChatGPT did not have information regarding references, citations, funding disclosures or dates of publication to verify this information.

### Readability assessment

The FRES score for the *Fact*‐based questions was 24.49, *Policy* questions was 22.82 and *Value* was 21.77. The FKGL for *Fact*‐based questions was 14.96, *Policy* was 14.78 and *Value* was 15.00. The FKGL number corresponds to the minimum reading level, indicating that the lowest grade level needed to read these answers would be Grade 15, or college‐graduate level.

## DISCUSSION

The purpose of this study is to analyze the quality, accessibility, and readability of information provided by ChatGPT (OpenAI, San Francisco) for commonly asked questions related to distal biceps tendon repair. Our search yielded DISCERN scores that were over 50, which is considered a ‘good’ score. The JAMA benchmark criteria were calculated to be 0 which indicates a lack of reliability. Finally, the low FRES values and a minimum of nearly college graduate level of reading for all the answers, based on our FKGL score, indicate complex answers. Importantly, all the answers provided by the AI model recommended reaching out to surgeons and medical teams to gain further education regarding more patient‐centred advice.

### Quality assessment

The DISCERN scores for all types of questions, *Fact, Policy and Value*, were considered ‘good’ scores, as they were all above 50. These scores were similar to scores cited in prior literature regarding shoulder stabilization surgery, rotator cuff repairs, shoulder arthroplasty and common hand procedures [3, 8, 21]. Most of these studies have not categorized questions by Rothwell criteria, and as such only reported one score for answers generated, aside from Warren et al. [21] who reported the highest score for the *Value* questions (55) and the lowest for the *Fact* questions (51). While there does not seem to be a distinct pattern of scores between the three categories, it is interesting to note that our study yielded the same score for *Fact* and *Value* questions (59), and higher score for *Policy* questions (61), although the difference in scoring is minimal. Overall, the quality of ChatGPT answers to distal biceps repair tends to be good, which is consistent with prior literature.

### Accuracy assessment

The JAMA benchmark criteria were 0 for *Fact, Policy and Value* questions as the answers provided by ChatGPT did not have information regarding references, citations, funding disclosures or dates of publication to verify this information. Therefore, these answers were given the lowest rating for all four criteria being measured. This trend of low JAMA benchmark scores has been noted in prior studies that have compared ChatGPT‐generated answers to website‐generated answers [8, 21]. In regard to online health information obtained when searching for distal biceps repair, Foster et al. [6] found a relatively low JAMA criteria score, but higher than our study (0.7) when analyzing information provided by video‐based sources such as YouTube. This indicates that even though ChatGPT generated answers that had good accuracy as measured by the DISCERN criteria, the reliability of the information provided can be questioned. This is especially concerning as prior studies have indicated that ChatGPT is additionally able to falsify information and references if requested [1, 5, 15, 22].

### Readability assessment

Both the low FRES scores (all under 30), and the high FKGL scores (all over 14), indicate that ChatGPT generated highly complex texts that required a minimum of grade 15 reading level, for the *Fact* (14.96), *Policy* (14.78) *and Value* (15.00) questions [5]. This high level of complexity of answers generated by ChatGPT supports prior shoulder‐focused studies, such as Warren et al. [21], which demonstrated a minimum reading level of 10th grade or higher for rotator cuff repair‐related questions. Hurley et al. [8] analyzed questions related to shoulder instability procedure and found a minimum reading level of a college graduate for the answers generated by ChatGPT. These studies have shown that ChatGPT‐generated answers continue to demonstrate higher complexity than is recommended by the National Institutes of Health (NIH) and the *American Medical Association* (sixth‐grade reading level) for medical information for patient education [25]. These results suggest that although the generated answers are appropriate in terms of content, readability may be a barrier for the average American who searches ChatGPT for answers related to distal biceps surgery.

There were limitations to our study. When obtaining our commonly asked questions, we modified questions from other common upper extremity injuries for distal biceps ruptures. We used this search as a surrogate for commonly asked distal biceps questions. The 27 questions were not selected based on a standardized search method and were collected using prior published studies related to shoulder pathologies. Two reviewers analyzed our questions prior to our search to limit questions that were out of scope of this study. It may be beneficial for ChatGPT to generate citations along with the answers, but this is not a current feature of ChatGPT; therefore, JAMA Benchmark will always be 0 when searching ChatGPT.

## CONCLUSIONS

The answers provided by ChatGPT were a good source of information in terms of quality assessment, compared to other online resources that also do not have citations as an option. The accuracy and reliability of these answers were shown to be low, with nearly a college‐graduate level of readability. This indicates that physicians should caution patients when searching ChatGPT for information regarding distal biceps repairs. ChatGPT serves as a promising source for patients to learn about their procedure, although its reliability and readability are disadvantages for the average patient when utilizing the software.

## AUTHOR CONTRIBUTIONS

Suhasini Gupta and Brett D. Haislup contributed to the conceptualization of the study and drafting of the manuscript. Ryan A. Hoffman and Anand M. Murthi provided critical revisions to the manuscript.

## CONFLICT OF INTEREST STATEMENT

The authors declare no conflicts of interest.

## ETHICS STATEMENT

The ethics statement is not available.

## Supporting information

Supporting information.

## Data Availability

Data sharing is not applicable to this article as no data sets were generated or analysed during the current study.
