# Supplementary material for: Assessing information provided via artificial intelligence regarding distal biceps tendon repair surgery
Source: J Exp Orthop. 2025 May 19;12(2):e70281. doi: 10.1002/jeo2.70281 (PMC12086791; doi:10.1002/jeo2.70281)
Supplement: Supplementary file 1 — Supporting information. [file JEO2-12-e70281-s001.docx]

**Appendix 1: AI software generated answers for questions regarding Distal Biceps Repair Surgery (by Rothwell Criteria):**

**Fact:**

1. How do you go to the bathroom after distal bicep repair surgery?

After distal biceps tendon repair surgery, the approach to going to the bathroom may depend on the specific instructions provided by your surgeon and the type of immobilization or restrictions in place. Here are some general tips that may be relevant, but it's crucial to follow your surgeon's guidance:

Assistance: In the initial days following surgery, you may need assistance from someone to help you with basic activities, including going to the bathroom. This could involve someone helping you stabilize your arm and providing support as needed.

Adaptive Equipment: Depending on your surgeon's recommendations, you may use adaptive equipment such as a raised toilet seat or a commode chair to make the process more comfortable and manageable.

Immobilization Device: If your arm is immobilized in a splint, brace, or sling, you'll need to be cautious about how you position it. Your surgeon will provide guidance on whether you can remove the immobilization device temporarily during bathroom activities or if you need to keep it in place.

Positioning: Pay attention to your body positioning. It may be helpful to lean or shift your body in a way that minimizes strain on the repaired arm. Using your unaffected arm for support and balance can also be beneficial.

Hygiene: Ensure good hygiene practices. If your arm is in a sling or brace, take care not to compromise the surgical site while cleaning yourself.

Communication with Healthcare Team: If you have concerns or questions about managing daily activities, including bathroom use, don't hesitate to communicate with your healthcare team. They can provide specific guidance based on your individual case. Always follow the postoperative instructions provided by your surgeon, as they will have the best understanding of your condition and the necessary precautions for a successful recovery. If you experience any unexpected issues or challenges, contact your healthcare provider promptly for guidance.

*JAMA Score: 0*

*Flesch-Kincaid Reading Ease Score: 38.08, Interpretation: Difficult*

*Flesch-Kincaid Grade Level: 13.03, Reading level: College*

The JAMA score is 0 out of 4, as the answer does not include any information about authorship, citations/references, disclosures, or dates for when this information was last reviewed or posted. The FRES score is 38.08 as per the formula outlined in the methods section, and as such a ‘Difficult’ text [6]. The FKGL score is 13.03, as per the formula outlined in the methods section, and as such a minimum of 14^th^ grade level is required to read the same, which makes this text at a College reading level.

1. Can you play sports after distal bicep repair surgery?

Returning to sports after distal biceps tendon repair surgery depends on various factors, including the type of surgery performed, the individual's overall health, the specific sport, and the rehabilitation progress. Here are some general considerations:

Rehabilitation Period: There is typically a period of rehabilitation after distal biceps tendon repair surgery. This phase focuses on gradually restoring strength, range of motion, and function to the repaired tendon. The length of this rehabilitation period varies but can range from several weeks to a few months.

Surgeon's Recommendations: Follow your surgeon's recommendations and guidelines for returning to physical activities, including sports. They will provide specific timelines and criteria based on your individual case.

Type of Sport: The type of sport you wish to engage in matters. Some sports may involve more intense and repetitive use of the arms, which could impact the healing process. Contact sports or those with a high risk of falls may require additional caution.

Gradual Return: It's essential to have a gradual and phased return to sports. Rushing back too quickly can increase the risk of re-injury. Start with low-impact activities and gradually progress to more demanding exercises as advised by your healthcare team.

Strength and Stability: Ensure that the repaired tendon has regained sufficient strength and stability before returning to sports. Your physical therapist will guide you through exercises aimed at strengthening the biceps and the surrounding muscles.

Clearance from Surgeon: Before resuming sports, it's important to obtain clearance from your surgeon. They will assess your progress, evaluate your range of motion, strength, and overall recovery to determine when it is safe to return to sports.

Listen to Your Body: Pay attention to how your body responds to activity. If you experience pain, discomfort, or any unusual sensations, it's crucial to communicate with your healthcare team and adjust your activities accordingly. It's important to note that each individual's recovery is unique, and the decision to return to sports should be based on a thorough evaluation by your healthcare team. Always prioritize your long-term health and follow the guidance provided by your surgeon, physical therapist, and other healthcare professionals involved in your care.

*JAMA Score: 0*

*Flesch-Kincaid Reading Ease Score: 34.49, Interpretation: Difficult*

*Flesch-Kincaid Grade Level: 12.85, Reading level: College*

The JAMA score is 0 out of 4, as the answer does not include any information about authorship, citations/references, disclosures, or dates for when this information was last reviewed or posted. The FRES score is 34.49 as per the formula outlined in the methods section, and as such a ‘Difficult’ text [6]. The FKGL score is 12.85, as per the formula outlined in the methods section, and as such a minimum of 13^th^ grade level is required to read the same, which makes this text at a College reading level.

1. What is the success rate of distal bicep repair surgery?

The success rate of distal biceps tendon repair surgery is generally high, with many patients experiencing positive outcomes and restored function. However, the success of the surgery can depend on various factors, including the individual patient, the severity of the injury, the surgical technique used, and adherence to postoperative rehabilitation. Success in distal biceps repair is often defined by factors such as:

Restored Strength and Function: A successful surgery should result in the restoration of strength and function in the affected arm. This is crucial for individuals who engage in activities that require arm strength, such as lifting and sports.

Minimal Complications: The absence of significant complications, such as infection or re-rupture, contributes to the success of the surgery.

Patient Satisfaction: The overall satisfaction of the patient with the outcome of the surgery is an important measure of success. This includes factors such as pain relief, range of motion, and the ability to return to normal activities.

The success rate can vary based on the surgical technique employed (e.g., single-incision or double-incision repair) and the patient's commitment to postoperative rehabilitation. In general, following the surgeon's postoperative guidelines and engaging in physical therapy can significantly contribute to a successful outcome. It's important to note that individual cases may vary, and success rates are not uniform across all patients. Factors such as pre-existing medical conditions, age, and the presence of other injuries can also influence the overall outcome. If you are considering distal biceps tendon repair surgery or have undergone the procedure, it's crucial to discuss your specific case with your orthopedic surgeon. They can provide personalized information regarding the expected outcomes, potential risks, and the rehabilitation process based on your individual circumstances.

*JAMA Score: 0*

*Flesch-Kincaid Reading Ease Score: 18.10, Interpretation: Very Difficult*

*Flesch-Kincaid Grade Level: 16.10, Reading level: College graduate*

The JAMA score is 0 out of 4, as the answer does not include any information about authorship, citations/references, disclosures, or dates for when this information was last reviewed or posted. The FRES score is 18.10 as per the formula outlined in the methods section, and as such a ‘Very Difficult’ text [6]. The FKGL score is 16.10, as per the formula outlined in the methods section, and as such a minimum of 17^th^ grade level is required to read the same, which makes this text at a College graduate reading level.

1. What are the most common complications of a distal bicep repair surgery?

Distal biceps tendon repair surgery is generally considered a safe and effective procedure, but like any surgery, it carries some potential risks and complications. The likelihood of complications can vary among individuals, and factors such as the nature of the injury, surgical technique, and postoperative care play roles in the overall outcome. Some common complications associated with distal biceps repair surgery include:

Infection: Infections at the surgical site are a potential complication. Signs of infection may include increased redness, swelling, warmth, or drainage from the incision. Infections can usually be managed with appropriate medical care.

Nerve Injury: Nerve injury is a rare but possible complication. It can result in symptoms such as numbness, tingling, or weakness in the affected arm. The risk is often minimized by careful surgical technique, but nerve irritation can occur.

Stiffness and Reduced Range of Motion: Some individuals may experience stiffness in the elbow or reduced range of motion after surgery. Physical therapy is crucial in preventing and addressing these issues.

Tendon Re-rupture: While uncommon, there is a risk of re-rupture of the repaired tendon. Adhering to postoperative restrictions and rehabilitation guidelines is essential to minimize this risk.

Heterotopic Ossification: In some cases, the formation of abnormal bone within the soft tissues (heterotopic ossification) may occur. This can potentially affect range of motion and may require additional treatment.

Pain Persistency: Some patients may experience persistent pain or discomfort after surgery. This can be due to various factors, including incomplete healing, nerve irritation, or other issues.

Complications Related to Anesthesia: Anesthesia-related complications, while rare, can occur. These may include allergic reactions, respiratory issues, or adverse reactions to anesthesia medications.

Hardware-related Issues: If hardware such as screws or anchors is used during the surgery, there can be complications related to the hardware, such as irritation, loosening, or the need for removal.

It's important for patients to discuss potential complications with their orthopedic surgeon before the surgery and to follow all postoperative care instructions. Surgeons take steps to minimize risks, and adherence to rehabilitation guidelines is crucial for a successful recovery. If you experience signs of infection, unusual pain, or any concerning symptoms after surgery, it's important to contact your healthcare provider promptly for further evaluation and appropriate management.

*JAMA Score: 0*

*Flesch-Kincaid Reading Ease Score: 20.79, Interpretation: Very Difficult*

*Flesch-Kincaid Grade Level: 14.54, Reading level: College*

The JAMA score is 0 out of 4, as the answer does not include any information about authorship, citations/references, disclosures, or dates for when this information was last reviewed or posted. The FRES score is 20.79 as per the formula outlined in the methods section, and as such a ‘Very Difficult’ text [6]. The FKGL score is 14.54, as per the formula outlined in the methods section, and as such a minimum of 15^th^ grade level is required to read the same, which makes this text at a College reading level.

1. What can you not do after distal bicep repair surgery?

After distal biceps tendon repair surgery, there are certain activities and movements that are typically restricted during the initial stages of recovery to protect the repaired tendon and promote healing. It's crucial to follow your surgeon's specific guidelines, as restrictions can vary based on the surgical technique used and individual patient factors. Here are some common precautions and activities to avoid:

Heavy Lifting: Avoid lifting heavy objects, as this can put excessive strain on the healing tendon. The weight restrictions will be provided by your surgeon and may vary during different phases of recovery.

Forceful Flexion and Extension: Refrain from forceful flexion (bending) and extension (straightening) of the elbow beyond the limits specified by your surgeon. This is important to prevent stress on the repaired tendon.

Rotational Movements: Limit rotational movements of the forearm, as these can also stress the healing tendon. Avoid activities that involve twisting or supination/pronation of the forearm.

Resisting Force: Avoid activities that involve resisting force with the affected arm. This includes pushing or pulling heavy objects.

Excessive Stretching: Refrain from performing excessive stretching exercises that may strain the repaired tendon. Follow the prescribed range of motion exercises provided by your physical therapist.

Impact Activities: Steer clear of high-impact activities or sports that could increase the risk of falls, collisions, or direct trauma to the healing area.

Unsupported Arm Positioning: Be cautious about unsupported arm positioning, especially in situations where the arm might be hanging or dangling without support.

Abrupt Movements: Avoid sudden and abrupt movements that could place stress on the healing tendon. Movements should be controlled and within the prescribed range.

Driving (initially): Driving may be restricted initially, especially if the surgical arm is the dominant arm. Consult with your surgeon regarding when it is safe to resume driving.

Skipping Physical Therapy: Attend all recommended physical therapy sessions and follow the prescribed exercises to regain strength and flexibility gradually.

Always communicate openly with your healthcare team and adhere to their instructions to ensure a successful recovery. As you progress through the rehabilitation process, your surgeon and physical therapist may modify restrictions and provide guidance on gradually reintroducing activities based on your individual recovery milestones.

*JAMA Score: 0*

*Flesch-Kincaid Reading Ease Score: 27.05, Interpretation: Very Difficult*

*Flesch-Kincaid Grade Level: 13.75, Reading level: College*

The JAMA score is 0 out of 4, as the answer does not include any information about authorship, citations/references, disclosures, or dates for when this information was last reviewed or posted. The FRES score is 27.05 as per the formula outlined in the methods section, and as such a ‘Very Difficult’ text [6]. The FKGL score is 13.75, as per the formula outlined in the methods section, and as such a minimum of 14^th^ grade level is required to read the same, which makes this text at a College reading level.

1. When can I begin driving after a distal bicep repair surgery?

The timing for resuming driving after distal biceps tendon repair surgery depends on several factors, including the specific details of the surgery, your overall recovery progress, and your ability to safely operate a vehicle. In general, it is recommended to wait until you have achieved sufficient mobility, strength, and control in the repaired arm before returning to driving. Here are some considerations regarding when you can resume driving after distal biceps tendon repair surgery:

Medical Clearance: Before resuming driving, it's important to obtain medical clearance from your surgeon. Your surgeon will assess your overall recovery, arm function, and any potential restrictions that may affect your ability to drive safely.

Arm Mobility and Strength: The ability to control and maneuver the vehicle is crucial for safe driving. You should have sufficient range of motion, strength, and coordination in the repaired arm to handle the steering wheel and other controls.

Comfort and Stability: You should feel comfortable and stable while sitting and operating the vehicle. Any pain, discomfort, or restrictions in movement that could impact your ability to drive safely should be addressed before getting back behind the wheel.

Use of Medications: If you are taking pain medications or other medications that may affect your alertness or coordination, it's essential to discuss this with your healthcare provider before driving.

Adherence to Postoperative Guidelines: Follow your surgeon's postoperative care guidelines, including any restrictions on lifting, arm movements, and physical activities. Adhering to these guidelines is crucial for the success of your recovery.

Gradual Return to Activities: Consider starting with short, local drives to assess your comfort and capability before attempting longer trips. Always consult with your surgeon before making decisions about when to resume driving. They can provide personalized guidance based on your specific case, the surgical approach used, and your individual recovery progress. It's important to prioritize safety and follow the recommendations of your healthcare team to avoid any potential risks associated with early resumption of driving.

*JAMA Score: 0*

*Flesch-Kincaid Reading Ease Score: 23.97, Interpretation: Very Difficult*

*Flesch-Kincaid Grade Level: 15.29, Reading level: College*

The JAMA score is 0 out of 4, as the answer does not include any information about authorship, citations/references, disclosures, or dates for when this information was last reviewed or posted. The FRES score is 23.97 as per the formula outlined in the methods section, and as such a ‘Very Difficult’ text [6]. The FKGL score is 15.29, as per the formula outlined in the methods section, and as such a minimum of 16^th^ grade level is required to read the same, which makes this text at a College reading level.

1. Can I take my splint off to sleep after distal bicep repair surgery?

Whether you can take off your splint to sleep after distal biceps tendon repair surgery depends on your surgeon's specific instructions and the type of splint used. In some cases, surgeons may allow patients to remove the splint while sleeping, especially as the initial healing period progresses. However, it's crucial to follow your surgeon's recommendations closely to ensure a successful recovery. Here are some general points to consider:

Early Postoperative Period: In the early postoperative period, surgeons often prescribe a splint or brace to immobilize the repaired arm and protect the healing tendon. During this phase, you may be instructed to wear the splint continuously, including while sleeping.

Progressive Changes: As you progress through the initial stages of recovery, your surgeon may modify the postoperative instructions. This may include allowing you to remove the splint for specific activities or periods, such as during sleep.

Nighttime Comfort: Wearing a splint at night can provide additional support and prevent inadvertent movements that may occur during sleep. However, if your surgeon allows it and you find that sleeping without the splint is comfortable and does not compromise the healing process, it may be permissible. It's important to communicate with your healthcare team and adhere to their recommendations. If you have questions about wearing the splint while sleeping or need clarification on specific instructions, contact your surgeon for guidance. They can provide personalized advice based on your individual case, the surgical technique used, and your progress in the recovery process. Remember that proper postoperative care, including adherence to splinting guidelines, is essential for the success of distal biceps tendon repair surgery.

*JAMA Score: 0*

*Flesch-Kincaid Reading Ease Score: 31.99, Interpretation: Difficult*

*Flesch-Kincaid Grade Level: 13.89, Reading level: College*

The JAMA score is 0 out of 4, as the answer does not include any information about authorship, citations/references, disclosures, or dates for when this information was last reviewed or posted. The FRES score is 31.99 as per the formula outlined in the methods section, and as such a ‘Difficult’ text [6]. The FKGL score is 13.89, as per the formula outlined in the methods section, and as such a minimum of 14^th^ grade level is required to read the same, which makes this text at a College reading level.

1. Can I have an MRI after distal bicep repair surgery?

The ability to have an MRI (Magnetic Resonance Imaging) after distal biceps tendon repair surgery may depend on several factors, and it's important to consult with your surgeon before scheduling any imaging procedures. Here are some considerations:

Type of Repair: The method used for distal biceps tendon repair can influence the decision to undergo an MRI. In some cases, certain repair techniques or implants used during surgery may have specific guidelines regarding MRI compatibility.

Timing of the MRI: The timing of the MRI in relation to your surgery is crucial. In the immediate postoperative period, there may be restrictions on certain activities, including undergoing an MRI, to allow for initial healing.

Consultation with Surgeon: Always consult with your orthopedic surgeon before scheduling an MRI. They can provide guidance based on your individual case, the specific surgical technique used, and the current status of your recovery.

Alternative Imaging Modalities: Depending on the clinical need for imaging and the restrictions in place, your healthcare team may consider alternative imaging modalities that do not pose the same risks or limitations as an MRI. Communication with Radiologist: If an MRI is deemed necessary, your surgeon may communicate with the radiologist to provide information about the surgery and any specific considerations related to the distal biceps repair. It's crucial to communicate openly with your healthcare team to ensure that any diagnostic procedures, including MRIs, are conducted safely and do not pose a risk to the healing process. Avoid scheduling any imaging procedures without consulting your surgeon, as they can provide tailored advice based on your unique circumstances

*JAMA Score: 0*

*Flesch-Kincaid Reading Ease Score: 19.92, Interpretation: Very Difficult*

*Flesch-Kincaid Grade Level: 16.39, Reading level: College Graduate*

The JAMA score is 0 out of 4, as the answer does not include any information about authorship, citations/references, disclosures, or dates for when this information was last reviewed or posted. The FRES score is 19.92 as per the formula outlined in the methods section, and as such a ‘Very Difficult’ text [6]. The FKGL score is 16.39 as per the formula outlined in the methods section, and as such a minimum of 17^th^ grade level is required to read the same, which makes this text at a College graduate reading level.

1. Where is the incision for distal bicep repair surgery?

The incision for distal biceps tendon repair surgery is typically made on the anterior (front) aspect of the elbow. There are two main approaches used for this surgery, each with its own incision location:

Single-Incision Approach: In a single-incision approach, the surgeon makes a single, larger incision on the anterior aspect of the elbow. The incision is generally made over the bicipital tuberosity, which is the prominence of the radius bone where the distal biceps tendon attaches. This approach provides direct access to the torn or ruptured tendon for repair.

Double-Incision Approach: In a double-incision approach, two smaller incisions are made: one at the front of the elbow and another on the forearm. The first incision at the elbow is used for visualization and repair of the tendon, while the second incision on the forearm is used for threading the tendon back through the bone. This approach may be chosen based on the surgeon's preference or the specific characteristics of the injury.

The choice between a single or double-incision approach depends on various factors, including the nature of the injury, surgeon's preference, and the patient's anatomy. Surgeons will carefully plan the incision(s) to optimize access to the damaged tendon while minimizing scarring and preserving the surrounding structures. It's important to discuss the specific surgical approach and incision details with your orthopedic surgeon before the procedure, as they can provide information tailored to your individual case. Following surgery, proper care of the incision site is essential for optimal healing and to minimize the risk of complications.

*JAMA Score: 0*

*Flesch-Kincaid Reading Ease Score: 29.30, Interpretation: Very Difficult*

*Flesch-Kincaid Grade Level: 14.92, Reading level: College*

The JAMA score is 0 out of 4, as the answer does not include any information about authorship, citations/references, disclosures, or dates for when this information was last reviewed or posted. The FRES score is 29.30 as per the formula outlined in the methods section, and as such a ‘Very Difficult’ text [6]. The FKGL score is 14.92, as per the formula outlined in the methods section, and as such a minimum of 15^th^ grade level is required to read the same, which makes this text at a College reading level.

1. How long should you take off work after distal bicep repair surgery?

The duration of time you should take off work after distal biceps tendon repair surgery can vary based on several factors, including the nature of your job, the type of surgery performed, and individual healing factors. It's crucial to follow your surgeon's recommendations and consider the specific demands of your occupation. Here are some general guidelines, but remember to consult with your surgeon for personalized advice:

Office or Sedentary Work: If your job involves primarily office or sedentary work, you may be able to return to work sooner, typically within a couple of weeks. Your surgeon may recommend modifications, such as avoiding heavy lifting or repetitive arm movements, during the initial period.

Manual or Labor-Intensive Work: Jobs that require manual labor or heavy lifting may require a more extended period off work. It's not uncommon for individuals with physically demanding jobs to take 6 weeks or more off work to allow for adequate healing and rehabilitation.

Surgeon's Recommendations: Follow your surgeon's specific recommendations regarding the duration of time off work. They will consider the specific details of your surgery, your overall health, and the physical demands of your job.

Physical Therapy and Rehabilitation: The timing of your return to work may also depend on your progress in physical therapy and rehabilitation. Your surgeon and physical therapist will assess your strength, range of motion, and overall recovery before providing clearance to return to work.

Gradual Return: In many cases, the return to work is a gradual process. Initially, you may have restrictions on certain activities, and your workload may be modified to accommodate your recovery. Always communicate openly with your healthcare team about your job requirements and any challenges you may face during the return-to-work process. It's essential to prioritize your recovery and follow the guidelines provided by your surgeon to ensure a successful outcome. If you have specific concerns or questions about returning to work, discuss them with your healthcare team for personalized advice.

*JAMA Score: 0*

*Flesch-Kincaid Reading Ease Score: 27.19, Interpretation: Very Difficult*

*Flesch-Kincaid Grade Level: 14.83, Reading level: College*

The JAMA score is 0 out of 4, as the answer does not include any information about authorship, citations/references, disclosures, or dates for when this information was last reviewed or posted. The FRES score is 27.19 as per the formula outlined in the methods section, and as such a ‘Very Difficult’ text [6]. The FKGL score is 14.83 as per the formula outlined in the methods section, and as such a minimum of 15^th^ grade level is required to read the same, which makes this text at a College reading level.

1. How long does it take to recover from a distal bicep repair surgery?

The recovery time after distal biceps tendon repair surgery can vary depending on several factors, including the specific surgical technique, individual patient characteristics, and how well postoperative instructions are followed. Here is a general timeline for recovery, but it's important to note that individual experiences may vary:

Immediate Postoperative Period (Weeks 1-2): During the first few weeks after surgery, the emphasis is on protecting the repaired tendon. The arm may be immobilized in a sling or brace, and restrictions on movement and activities are typically in place. Physical therapy may begin to focus on gentle range of motion exercises.

Early Rehabilitation (Weeks 2-6): The initial immobilization phase may be gradually transitioned to more active rehabilitation. Physical therapy becomes more intensive, focusing on restoring range of motion, reducing swelling, and preventing stiffness. Light strengthening exercises may be introduced, but heavy lifting is generally avoided.

Mid-Recovery (Weeks 6-12): Strengthening exercises become more challenging and are progressively increased. The emphasis is on regaining muscle strength and endurance in the affected arm. Return to normal daily activities is encouraged, but certain restrictions on heavy lifting and high-impact activities may still be in place.

Late Recovery (Months 3-6 and Beyond): During this phase, most individuals experience significant improvement in strength and function. The focus shifts toward achieving full strength, stability, and a gradual return to more demanding activities. Final assessments by the surgeon and physical therapist may guide the decision on when to resume sports or activities with higher physical demands.

It's important to note that the above timeline is a general overview, and the specific details of your recovery will be influenced by your surgeon's recommendations, the surgical technique used, and your individual progress. Compliance with postoperative rehabilitation, including attending physical therapy sessions and following home exercise programs, is crucial for a successful recovery. Always communicate openly with your healthcare team about your progress, and follow their guidance regarding activity levels and restrictions to ensure optimal healing and long-term outcomes. If you have specific questions about your recovery, consult with your orthopedic surgeon or physical therapist.

*JAMA Score: 0*

*Flesch-Kincaid Reading Ease Score: 15.24, Interpretation: Very Difficult*

*Flesch-Kincaid Grade Level: 16.13, Reading level: College graduate*

The JAMA score is 0 out of 4, as the answer does not include any information about authorship, citations/references, disclosures, or dates for when this information was last reviewed or posted. The FRES score is 15.24 as per the formula outlined in the methods section, and as such a ‘Very Difficult’ text [6]. The FKGL score is 16.13 as per the formula outlined in the methods section, and as such a minimum of 17^th^ grade level is required to read the same, which makes this text at a College graduate reading level.

1. How long is physical therapy after a distal bicep repair surgery?

The duration of physical therapy after distal biceps tendon repair surgery varies from patient to patient and is influenced by factors such as the surgical technique, individual healing, and overall progress. Physical therapy is typically a crucial component of the rehabilitation process, helping to restore strength, range of motion, and function in the affected arm. Here are some general guidelines for the duration of physical therapy after distal biceps repair:

Immediate Postoperative Period (Weeks 1-2): Physical therapy often begins shortly after surgery, focusing initially on gentle range of motion exercises and managing swelling. The primary goal is to prevent stiffness and encourage early movement within the limits set by the surgeon.

Early Rehabilitation (Weeks 2-6): During this phase, physical therapy becomes more active, with a focus on increasing range of motion, reducing swelling, and addressing any muscle imbalances. Gentle strengthening exercises may be introduced, and the rehabilitation program is tailored to the individual's progress.

Mid-Recovery (Weeks 6-12): Strengthening exercises become more challenging and targeted. The emphasis is on regaining muscle strength, stability, and function in the repaired arm. Specific exercises may be designed to mimic functional activities and prepare the individual for a gradual return to normal daily activities.

Late Recovery (Months 3-6 and Beyond): Physical therapy may continue during this phase, focusing on advanced strengthening, coordination, and functional activities. The duration of physical therapy sessions may decrease, and the emphasis shifts toward independence in maintaining the gains achieved during rehabilitation. The overall duration of physical therapy can vary, but it is not uncommon for individuals to participate in a formal physical therapy program for approximately 3 to 6 months. The frequency of sessions may be more frequent in the early stages and gradually decrease as the individual progresses.

It's important to note that these timelines are general estimates, and the specific plan will be individualized based on factors such as the patient's response to therapy, the nature of the surgery, and any additional considerations. Compliance with the physical therapy program and ongoing communication with the healthcare team are essential for a successful recovery. If you have questions or concerns about your physical therapy plan, discuss them with your physical therapist and orthopedic surgeon.

*JAMA Score: 0*

*Flesch-Kincaid Reading Ease Score: 14.77, Interpretation: Very Difficult*

*Flesch-Kincaid Grade Level: 16.85, Reading level: College graduate*

The JAMA score is 0 out of 4, as the answer does not include any information about authorship, citations/references, disclosures, or dates for when this information was last reviewed or posted. The FRES score is 14.77 as per the formula outlined in the methods section, and as such a ‘Very Difficult’ text [6]. The FKGL score is 16.85 as per the formula outlined in the methods section, and as such a minimum of 17^th^ grade level is required to read the same, which makes this text at a College graduate reading level.

1. What is the average age for a distal bicep repair surgery?

The decision to undergo distal biceps tendon repair surgery is not solely determined by age but rather by the specific circumstances of the injury and the individual's overall health. Distal biceps tendon injuries can occur in individuals of various ages, and surgical intervention may be considered based on factors such as the severity of the injury, functional impairment, and the individual's goals and lifestyle. That said, distal biceps tendon injuries are more commonly seen in individuals who are active, engage in weightlifting, or participate in activities that involve repetitive or forceful use of the arms. These injuries can occur in both younger and older adults. The average age for distal biceps repair surgery can vary, but it is often seen in adults between the ages of 30 and 60. However, individuals outside this age range may also undergo the surgery based on their specific circumstances. Ultimately, the decision for surgery is made on a case-by-case basis after a thorough evaluation by an orthopedic surgeon. Factors such as the patient's overall health, functional goals, and the nature of the injury will be considered in determining the appropriate course of treatment. If you suspect a distal biceps tendon injury or are considering surgery, it's important to consult with an orthopedic specialist who can assess your individual case and provide personalized recommendations based on your unique circumstances.

*JAMA Score: 0*

*Flesch-Kincaid Reading Ease Score: 23.45, Interpretation: Very Difficult*

*Flesch-Kincaid Grade Level: 15.68, Reading level: College*

The JAMA score is 0 out of 4, as the answer does not include any information about authorship, citations/references, disclosures, or dates for when this information was last reviewed or posted. The FRES score is 23.45 as per the formula outlined in the methods section, and as such a ‘Very Difficult’ text [6]. The FKGL score is 15.68 as per the formula outlined in the methods section, and as such a minimum of 16^th^ grade level is required to read the same, which makes this text at a College reading level.

1. Can you throw a ball after distal bicep repair surgery?

The ability to throw a ball after distal biceps tendon repair surgery depends on various factors, including the specific details of the surgery, the individual's recovery progress, and the guidance of the orthopedic surgeon. Here are some general considerations:

Postoperative Restrictions: In the initial stages of recovery, there are typically restrictions on certain movements and activities to protect the repaired tendon. Your surgeon will provide specific guidelines on what is allowed and what should be avoided.

Rehabilitation and Physical Therapy: Rehabilitation and physical therapy play a crucial role in the recovery process. The gradual reintroduction of activities, including throwing motions, will be guided by your physical therapist and surgeon.

Timing and Progression: The timing of when you can start throwing a ball will depend on how well the tendon heals and your progression in the rehabilitation program. It is a gradual process, and rushing back to activities too soon can increase the risk of re-injury.

Individual Variability: Each individual's recovery is unique, and the ability to throw a ball will vary based on factors such as the type of surgery, overall health, and adherence to rehabilitation protocols.

Surgeon's Clearance: Before engaging in activities like throwing a ball, it's important to obtain clearance from your orthopedic surgeon. They will assess your recovery, range of motion, and strength before giving the green light for more dynamic activities. Always follow the specific postoperative instructions provided by your surgeon and communicate openly with your healthcare team about your progress. If throwing a ball is part of your regular activities or sports, discuss this with your surgeon and physical therapist to ensure a safe and gradual return to such motions. Attempting to throw a ball too early or without proper guidance can jeopardize the success of the surgery and may lead to complications.

*JAMA Score: 0*

*Flesch-Kincaid Reading Ease Score: 23.45*

*Flesch-Kincaid Grade Level: 15.68, College level*

The JAMA score is 0 out of 4, as the answer does not include any information about authorship, citations/references, disclosures, or dates for when this information was last reviewed or posted. The FRES score is 23.45 as per the formula outlined in the methods section, and as such a ‘Very Difficult’ text [6]. The FKGL score is 15.68 as per the formula outlined in the methods section, and as such a minimum of 16^th^ grade level is required to read the same, which makes this text at a College reading level.

1. How much does Medicare pay for distal bicep repair surgery?

The specific amount that Medicare pays for distal biceps tendon repair surgery can vary based on several factors, including the type of Medicare coverage, the specific details of the surgery, and any applicable deductibles or coinsurance. Medicare typically consists of different parts, each covering different aspects of healthcare services:

Medicare Part A: This primarily covers hospital and inpatient services. If distal biceps repair surgery is performed on an inpatient basis, Part A may cover certain costs associated with the hospital stay.

Medicare Part B: This covers outpatient services, including surgeries performed in outpatient settings such as ambulatory surgical centers or physician's offices. Part B typically covers physician services, outpatient hospital services, and certain other healthcare services.

Medicare Advantage (Part C): Some individuals choose to enroll in Medicare Advantage plans offered by private insurance companies. These plans, while required to cover at least the same services as original Medicare, may have different cost-sharing structures.

Medicare Part D: This part covers prescription drug costs, and it is not directly related to surgical procedures.

The specific costs and coverage details for distal biceps repair surgery under Medicare will depend on whether the procedure is performed in an inpatient or outpatient setting, the provider's billing practices, and any additional services or treatments required. It's important to check with Medicare or your Medicare Advantage plan for the most accurate and up-to-date information on coverage and costs. Additionally, contacting the healthcare provider or facility where the surgery is planned can provide information on specific billing and cost details related to the procedure. Keep in mind that deductibles, coinsurance, and other out-of-pocket costs may apply, so understanding the full financial picture is essential.

*JAMA Score: 0*

*Flesch-Kincaid Reading Ease Score: 16.74, Interpretation: Very Difficult*

*Flesch-Kincaid Grade Level: 16.64, Reading level: College graduate*

The JAMA score is 0 out of 4, as the answer does not include any information about authorship, citations/references, disclosures, or dates for when this information was last reviewed or posted. The FRES score is 16.74 as per the formula outlined in the methods section, and as such a ‘Very Difficult’ text [6]. The FKGL score is 16.64 as per the formula outlined in the methods section, and as such a minimum of 17^th^ grade level is required to read the same, which makes this text at a College graduate reading level.

The DISCERN scores for all the *Fact* questions was 61, which is interpretated as a good source of information. In terms of reliability, these 15 questions are answered with adequate information provided by ChatGPT, referrers to uncertainty in all the answers, but lacks any information regarding sources, and additional sources of information. The overall quality of information provided about the treatment choices ranges from adequate to excellent, and as such the score for Section 2 and Section 3 of the DISCERN criteria is higher.

**Policy:**

1. Can you wait too long for distal bicep repair surgery?

While the timing of distal biceps tendon repair surgery is an important consideration, there is a general principle that earlier intervention is often preferred. Delaying surgery for a distal biceps tendon tear may have several implications:

Retraction of the Tendon: Over time, a torn distal biceps tendon may retract, making surgical repair more challenging. A retracted tendon may require more extensive procedures or grafts to reattach it properly.

Muscle Atrophy and Weakness: Without surgical intervention, the biceps muscle may undergo atrophy (loss of muscle mass) and weaken over time. This can affect the overall function of the arm.

Functional Limitations: Delaying surgery may lead to functional limitations, especially in activities that require strength and stability in the affected arm.

Scar Tissue Formation: In the absence of surgical repair, scar tissue may form around the torn tendon, potentially complicating the surgical procedure later on.

Risk of Further Injury: A partially torn or completely ruptured distal biceps tendon is at risk of further injury if not addressed promptly. Engaging in activities that stress the damaged tendon may increase the risk of complications.

While early intervention is generally preferred, the decision to undergo surgery is based on various factors, including the severity of the tear, the patient's overall health, and individual preferences. In some cases, conservative approaches such as physical therapy may be considered initially, especially for partial tears or cases with minimal symptoms. It's crucial to consult with an orthopedic surgeon to assess the specific details of the injury and determine the most appropriate course of action. The surgeon will consider factors such as the extent of the tear, the patient's lifestyle and goals, and the potential risks and benefits of surgery. Early consultation and timely intervention can contribute to a more successful outcome and minimize the potential challenges associated with delayed surgery.

*JAMA Score: 0*

*Flesch-Kincaid Reading Ease Score: 23.40, Interpretation: Very Difficult*

*Flesch-Kincaid Grade Level: 15.34, Reading level: College*

The JAMA score is 0 out of 4, as the answer does not include any information about authorship, citations/references, disclosures, or dates for when this information was last reviewed or posted. The FRES score is 23.40 as per the formula outlined in the methods section, and as such a ‘Very Difficult’ text [6]. The FKGL score is 15.34 as per the formula outlined in the methods section, and as such a minimum of 16^th^ grade level is required to read the same, which makes this text at a College reading level.

1. Are there alternatives to distal bicep repair surgery?

Yes, there are alternative approaches to distal biceps tendon repair surgery, especially in cases of partial tears or when surgery is not immediately necessary. The appropriate treatment depends on the extent of the injury, symptoms, and individual factors. Here are some alternatives to surgery:

Conservative Management: For partial tears or mild injuries, conservative management may be considered. This often involves rest, activity modification, and physical therapy. Physical therapy can help improve strength, flexibility, and function in the affected arm. It may include targeted exercises to strengthen the surrounding muscles and support the injured tendon.

Corticosteroid Injections: In some cases, corticosteroid injections may be used to reduce inflammation and alleviate pain associated with a distal biceps tendon injury. However, these injections are typically not recommended for complete tears.

Orthobiologics: Orthobiologics, such as platelet-rich plasma (PRP) or stem cell injections, may be considered as alternatives. These substances are derived from the patient's own blood or tissues and are believed to promote healing.

Bracing and Immobilization: Immobilizing the affected arm with a brace or splint may be recommended to allow the tendon to heal, especially in cases of partial tears. However, this approach may not be suitable for all types of injuries. It's important to note that while these alternatives may be considered, they are not always suitable for every case, especially in the presence of a complete or severe distal biceps tendon tear. Surgical intervention is often recommended for complete tears or cases where conservative measures are not effective. Individualized treatment decisions should be made in consultation with an orthopedic surgeon who can assess the specific details of the injury, consider the patient's goals and lifestyle, and provide personalized recommendations. If you suspect a distal biceps tendon injury, seeking prompt medical evaluation is crucial to determine the most appropriate course of action.

*JAMA Score: 0*

*Flesch-Kincaid Reading Ease Score: 22.55, Interpretation: Very Difficult*

*Flesch-Kincaid Grade Level: 14.77, Reading level: College*

The JAMA score is 0 out of 4, as the answer does not include any information about authorship, citations/references, disclosures, or dates for when this information was last reviewed or posted. The FRES score is 22.55 as per the formula outlined in the methods section, and as such a ‘Very Difficult’ text [6]. The FKGL score is 14.77 as per the formula outlined in the methods section, and as such a minimum of 15^th^ grade level is required to read the same, which makes this text at a College reading level.

1. What is the fastest way to recover from distal bicep repair surgery?

Recovery from distal biceps tendon repair surgery involves a gradual and structured process to ensure optimal healing and minimize the risk of complications. While there is no shortcut to a safe and complete recovery, there are steps you can take to support the healing process and potentially enhance your overall recovery experience:

Follow Postoperative Instructions: Adhere closely to the postoperative instructions provided by your surgeon. This includes guidelines on activities, range of motion, weight-bearing restrictions, and medication.

Attend Physical Therapy: Participate in a comprehensive physical therapy program as prescribed by your surgeon. Physical therapy is crucial for regaining strength, flexibility, and function in the repaired arm.

Manage Swelling: Follow any recommendations for managing swelling, such as elevating the arm and using cold compresses. Swelling management is important for both comfort and optimal healing.

Take Prescribed Medications: Take any prescribed medications, including pain relievers and anti-inflammatory drugs, as directed by your surgeon. This can help manage pain and reduce inflammation.

Engage in Gradual Rehabilitation: Gradually progress through rehabilitation exercises and avoid pushing yourself too hard too soon. Your physical therapist will guide you through a structured program tailored to your recovery.

Maintain a Healthy Lifestyle: Eat a balanced diet and stay hydrated to support overall health and healing. Adequate nutrition is essential for tissue repair.

Avoid Smoking: If you smoke, consider quitting or at least reducing smoking during the recovery period. Smoking can impair the healing process and increase the risk of complications.

Stay Active within Limits: Stay active within the limits set by your surgeon and physical therapist. Gentle movement can help prevent stiffness and promote circulation. Communicate with Your Healthcare Team: Keep open lines of communication with your healthcare team. Report any unusual symptoms, pain, or concerns promptly.

Patience and Rest: Allow yourself adequate rest and be patient with the recovery process. Avoid activities that are not cleared by your surgeon and follow the recommended timelines. It's important to note that every individual's recovery is unique, and the speed of recovery can vary. Attempting to rush the process may increase the risk of complications. Always consult with your surgeon and physical therapist for personalized advice and guidance based on your specific case and progress.

*JAMA Score: 0*

*Flesch-Kincaid Reading Ease Score: 29.38, Interpretation: Very Difficult*

*Flesch-Kincaid Grade Level: 12.94, Reading level: College*

The JAMA score is 0 out of 4, as the answer does not include any information about authorship, citations/references, disclosures, or dates for when this information was last reviewed or posted. The FRES score is 29.38 as per the formula outlined in the methods section, and as such a ‘Very Difficult’ text [6]. The FKGL score is 12.94 as per the formula outlined in the methods section, and as such a minimum of 13^th^ grade level is required to read the same, which makes this text at a College reading level.

1. How can I avoid distal bicep repair surgery?

Preventing a distal biceps tendon injury and the need for surgery involves adopting certain lifestyle practices and behaviors to reduce the risk of trauma and overuse. While it's not always possible to prevent injuries entirely, the following measures may help minimize the risk of distal biceps tendon injuries:

Strengthening Exercises: Incorporate regular strength training exercises that target the muscles around the elbow, including the biceps and forearm muscles. A well-balanced strength training program can enhance muscle strength and support joint stability.

Proper Lifting Techniques: Use proper lifting techniques to avoid putting excessive strain on the biceps tendon. Lift with your legs, keep the load close to your body, and avoid heavy lifting without proper form.

Avoid Overloading: Avoid excessive or repetitive heavy lifting, especially with activities like weightlifting, which can strain the biceps tendon. Gradually progress in weight and intensity to allow your muscles and tendons to adapt.

Warm-Up and Stretching: Prior to engaging in physical activities, warm up with dynamic exercises, and include stretching to improve flexibility. This can help prepare your muscles and tendons for the demands of exercise.

Cross-Training: Engage in a variety of physical activities and sports to prevent overuse of specific muscle groups. Cross-training can help distribute the workload and reduce the risk of repetitive strain.

Listen to Your Body: Pay attention to signs of fatigue, discomfort, or pain during physical activities. If you experience persistent pain, it's important to address it promptly and seek medical advice.

Use Protective Gear: When participating in sports or activities with a risk of impact or injury to the elbow, consider using protective gear, such as elbow pads or braces.

Stay Hydrated and Maintain Nutrition: Proper hydration and nutrition are essential for maintaining overall musculoskeletal health. Ensure you are well-hydrated and maintain a balanced diet rich in nutrients that support tendon and muscle health.

Gradual Progression: When starting a new exercise program or increasing intensity, progress gradually to allow your body to adapt and reduce the risk of overuse injuries.

Regular Check-Ups: If you have a history of elbow or biceps issues, consider regular check-ups with a healthcare professional to monitor and address any potential concerns early.

It's important to note that these measures can contribute to injury prevention, but they may not eliminate the risk entirely. If you experience persistent pain, weakness, or signs of a potential distal biceps tendon injury, consult with a healthcare professional for an accurate diagnosis and appropriate management.

*JAMA Score: 0*

*Flesch-Kincaid Reading Ease Score: 28.15, Interpretation: Very Difficult*

*Flesch-Kincaid Grade Level: 14.36, Reading level: College*

The JAMA score is 0 out of 4, as the answer does not include any information about authorship, citations/references, disclosures, or dates for when this information was last reviewed or posted. The FRES score is 28.15 as per the formula outlined in the methods section, and as such a ‘Very Difficult’ text [6]. The FKGL score is 14.36 as per the formula outlined in the methods section, and as such a minimum of 15^th^ grade level is required to read the same, which makes this text at a College reading level.

1. Is distal bicep repair surgery an inpatient or outpatient procedure?

The classification of distal biceps tendon repair surgery as an inpatient or outpatient procedure can depend on various factors, including the specific details of the surgery, the surgeon's preference, and the overall health of the patient. In many cases, distal biceps tendon repair is performed as an outpatient procedure. However, there are instances where it may be done on an inpatient basis.

Outpatient Procedure: Distal biceps tendon repair is often performed on an outpatient basis, meaning the patient undergoes surgery and is discharged on the same day. The outpatient setting could be an ambulatory surgical center or a hospital's outpatient surgery department.

Inpatient Procedure: In certain situations, such as when additional medical concerns or complexities are present, the surgery may be performed on an inpatient basis. Inpatient surgery involves the patient staying overnight in the hospital for postoperative monitoring and care. The choice between outpatient and inpatient surgery is influenced by factors such as the patient's overall health, the extent of the surgery, the surgeon's assessment, and institutional policies. Distal biceps tendon repair is commonly performed using minimally invasive techniques, contributing to the preference for outpatient procedures. It's important for patients to discuss the planned surgical approach, expected length of the procedure, and the anticipated recovery process with their orthopedic surgeon. The surgeon will provide information on whether the surgery will be performed as an outpatient or inpatient procedure based on the individual case. Additionally, patients will receive postoperative care instructions and information on follow-up appointments for monitoring the healing process.

*JAMA Score: 0*

*Flesch-Kincaid Reading Ease Score: 16.40, Interpretation: Very Difficult*

*Flesch-Kincaid Grade Level: 16.16, Reading level: College graduate*

The JAMA score is 0 out of 4, as the answer does not include any information about authorship, citations/references, disclosures, or dates for when this information was last reviewed or posted. The FRES score is 16.40 as per the formula outlined in the methods section, and as such a ‘Very Difficult’ text [6]. The FKGL score is 16.16 as per the formula outlined in the methods section, and as such a minimum of 17^th^ grade level is required to read the same, which makes this text at a College graduate reading level.

1. What is the chance of dying in distal bicep repair surgery?

Distal biceps tendon repair surgery is generally considered a safe and routine procedure with a low risk of serious complications, including the risk of death. However, as with any surgical procedure, there are inherent risks associated with anesthesia, surgery, and postoperative recovery. It's important to note that the overall risk of mortality is extremely low for elective orthopedic surgeries, and complications leading to death are rare. Factors that contribute to the low risk of mortality in distal biceps tendon repair surgery include:

Elective Nature: Distal biceps tendon repair is typically an elective, planned procedure. Elective surgeries generally have lower risks compared to emergency or urgent surgeries.

Minimally Invasive Techniques: Many distal biceps tendon repair surgeries are performed using minimally invasive techniques, which can contribute to a quicker recovery and reduced risk of complications.

Patient Selection: Surgeons carefully assess patients before recommending surgery. Preoperative evaluations help identify and manage any potential risk factors that could impact the safety of the procedure.

Advancements in Anesthesia: Modern anesthesia techniques and monitoring contribute to the safety of surgical procedures. Anesthesia providers monitor patients closely during surgery to ensure their well-being.

Postoperative Care: Adequate postoperative care and follow-up contribute to the overall safety of the procedure. Patients receive instructions for postoperative recovery and are monitored for any signs of complications.

While the risk of mortality is low, it's important for patients to discuss potential risks and complications with their orthopedic surgeon and anesthesia team before undergoing surgery. Individual risk factors, such as pre-existing health conditions, will be taken into consideration during the preoperative evaluation. As with any surgery, there can be risks of complications such as infection, bleeding, anesthesia-related issues, or problems specific to the surgical procedure. Patients are encouraged to have open and thorough discussions with their healthcare team to address any concerns and ensure they are well-informed about the procedure and its potential risks.

*JAMA Score: 0*

*Flesch-Kincaid Reading Ease Score: 11.37, Interpretation: Very Difficult*

*Flesch-Kincaid Grade Level: 16.50, Reading level: College graduate*

The JAMA score is 0 out of 4, as the answer does not include any information about authorship, citations/references, disclosures, or dates for when this information was last reviewed or posted. The FRES score is 11.37 as per the formula outlined in the methods section, and as such a ‘Very Difficult’ text [6]. The FKGL score is 16.50 as per the formula outlined in the methods section, and as such a minimum of 17^th^ grade level is required to read the same, which makes this text at a College graduate reading level.

The DISCERN scores for all the *Policy* questions was 61, which is interpretated as a good source of information. In terms of reliability, questions 16-21 are answered with adequate information provided by ChatGPT, referrers to uncertainty in all the answers, but lacks any information regarding sources, and additional sources of information. The overall quality of information provided about the treatment choices ranges from good to excellent, especially in regard to Section 2 of the DISCERN criteria, due to increased discussion regarding quality of life and different possible treatment options.

**Value:**

1. Why is distal bicep repair surgery so painful?

Distal biceps tendon repair surgery can be associated with postoperative pain, and the level of pain experienced can vary from person to person. Several factors contribute to the pain after surgery, and understanding these factors can help manage and alleviate discomfort. Here are some reasons why distal biceps repair surgery can be painful:

Surgical Trauma: The surgical procedure itself involves manipulating tissues, cutting, and reattaching the tendon. Surgical trauma to the soft tissues can result in pain during the initial recovery period.

Inflammation and Swelling: Surgery triggers an inflammatory response as part of the body's natural healing process. Inflammation and swelling in the surgical area can contribute to pain and discomfort.

Nerve Irritation: Manipulation of tissues during surgery can irritate nerves in the vicinity, leading to temporary nerve-related pain. Nerve irritation can cause sensations such as tingling, numbness, or shooting pain.

Recovery and Healing Process: The early stages of the healing process involve tissue repair and regeneration. As tissues heal, pain and discomfort are common. The repaired tendon needs time to integrate and regain strength.

Immobilization: Following surgery, the affected arm is often immobilized in a sling or brace to protect the repair. Immobilization can contribute to stiffness and discomfort during the initial recovery period.

Muscle Spasms: Muscle spasms may occur as a response to surgery and the altered biomechanics of the affected arm. Spasms can contribute to pain and tightness.

Individual Pain Perception: Pain is subjective, and individuals may have varying pain thresholds and perceptions. Factors such as pain tolerance, anxiety, and psychological factors can influence the experience of pain. To manage postoperative pain after distal biceps repair surgery, healthcare providers typically prescribe pain medications or recommend over-the-counter pain relievers.

Additionally, the following measures can help alleviate pain:

Elevating the Arm: Elevating the arm can reduce swelling and alleviate discomfort.

Icing the Surgical Area: Applying ice to the surgical area can help control inflammation and numb the area.

Physical Therapy: Gradual introduction of range of motion exercises and physical therapy can help prevent stiffness and improve overall recovery.

It's important for patients to communicate openly with their healthcare team about their pain levels, and healthcare providers can adjust pain management strategies based on individual needs. If pain persists or becomes severe, it's crucial to seek prompt medical attention for further evaluation.

*JAMA Score: 0*

*Flesch-Kincaid Reading Ease Score: 23.66, Interpretation: Very Difficult*

*Flesch-Kincaid Grade Level: 14.08, Reading level: College*

The JAMA score is 0 out of 4, as the answer does not include any information about authorship, citations/references, disclosures, or dates for when this information was last reviewed or posted. The FRES score is 23.66 as per the formula outlined in the methods section, and as such a ‘Very Difficult’ text [6]. The FKGL score is 14.08 as per the formula outlined in the methods section, and as such a minimum of 15^th^ grade level is required to read the same, which makes this text at a College reading level.

1. What happens if you don't wear your splint after distal bicep repair surgery?

Wearing a splint or brace after distal biceps tendon repair surgery is a critical component of the postoperative care plan. The splint is designed to provide support, protect the repaired tendon, and prevent movements that could jeopardize the healing process. Failing to wear the splint as prescribed by your surgeon can have various consequences, including:

Increased Risk of Re-injury: Without the support of the splint, the repaired distal biceps tendon may be exposed to excessive stress and strain. This increases the risk of re-injury, disrupting the healing process and potentially requiring additional interventions.

Compromised Surgical Outcome: The splint plays a crucial role in maintaining the correct positioning of the repaired tendon during the early stages of healing. Neglecting to wear the splint can compromise the overall surgical outcome and affect the success of the repair.

Delayed Healing: The splint helps to immobilize the affected arm, allowing the repaired tendon to heal properly. Lack of immobilization may lead to increased movement and delayed healing, prolonging the recovery process.

Increased Pain and Discomfort: The splint provides support and stability, contributing to pain control and reducing discomfort. Without the splint, patients may experience increased pain, swelling, and discomfort.

Risk of Complications: Non-compliance with postoperative instructions, including splint use, can increase the risk of complications such as tendon re-rupture, development of scar tissue, or issues related to improper healing. It's crucial to follow your surgeon's instructions regarding splint use and wear the splint for the recommended duration. Surgeon’s tailor postoperative care plans based on the specific details of the surgery, the patient's individual needs, and the chosen rehabilitation approach. If you have concerns about the splint, experience discomfort, or find it challenging to adhere to the prescribed guidelines, communicate openly with your healthcare team. They can provide guidance, address your concerns, and make any necessary adjustments to ensure that you are on track for a successful recovery. Adhering to the prescribed postoperative care plan is essential for achieving optimal outcomes after distal biceps tendon repair surgery.

*JAMA Score: 0*

*Flesch-Kincaid Reading Ease Score: 30.08, Interpretation: Difficult*

*Flesch-Kincaid Grade Level: 14.22, Reading level: College*

The JAMA score is 0 out of 4, as the answer does not include any information about authorship, citations/references, disclosures, or dates for when this information was last reviewed or posted. The FRES score is 30.08 as per the formula outlined in the methods section, and as such a ‘Difficult’ text [6]. The FKGL score is 14.22 as per the formula outlined in the methods section, and as such a minimum of 15^th^ grade level is required to read the same, which makes this text at a College reading level.

1. Can you ice too much after distal bicep repair surgery?

While icing is a common and effective method for managing pain and swelling after distal biceps tendon repair surgery, it is possible to ice too much or use improper techniques. Excessive or prolonged icing can lead to potential complications. Here are some considerations regarding icing after surgery:

Duration and Frequency: It's important to follow the recommended duration and frequency of icing as advised by your surgeon or healthcare provider. Applying ice for too long or too frequently may cause skin damage, frostbite, or other adverse effects.

Protecting the Skin: Always use a cloth or towel to protect the skin when applying ice. Direct contact between ice and the skin can result in cold injuries, including frostbite or skin irritation.

Avoiding Ice Pack Overuse: While ice packs can be beneficial for controlling swelling, it's important not to overuse them. Extended use of ice packs without breaks may lead to issues such as skin damage or nerve irritation.

Monitoring Skin Sensation: Pay attention to the sensation of the skin during and after icing. If you experience numbness, tingling, or discomfort, discontinue icing and allow the skin to return to normal temperature.

Balancing Cold Therapy with Other Methods: Cold therapy is just one component of postoperative care. It should be combined with other recommended strategies such as elevation, rest, and prescribed medications for comprehensive pain and swelling management.

Consulting with Healthcare Providers: If you have concerns about icing or are unsure about the appropriate use, consult with your surgeon or healthcare provider. They can provide specific guidance tailored to your individual case. Remember that proper postoperative care, including icing, is essential for a successful recovery. However, it's equally important to follow the specific instructions provided by your surgeon and to communicate any concerns or issues you may be experiencing. If you are unsure about the correct way to apply ice or have questions about your postoperative care, seek guidance from your healthcare team for personalized advice.

*JAMA Score: 0*

*Flesch-Kincaid Reading Ease Score: 34.59, Interpretation: Difficult*

*Flesch-Kincaid Grade Level: 13.15, Reading level: College*

The JAMA score is 0 out of 4, as the answer does not include any information about authorship, citations/references, disclosures, or dates for when this information was last reviewed or posted. The FRES score is 34.59 as per the formula outlined in the methods section, and as such a ‘Difficult’ text [6]. The FKGL score is 13.15 as per the formula outlined in the methods section, and as such a minimum of 14^th^ grade level is required to read the same, which makes this text at a College reading level.

1. How painful is distal bicep repair surgery?

Distal biceps tendon repair surgery is typically associated with some level of postoperative pain, as with many surgical procedures. However, pain experiences can vary widely among individuals, and factors such as pain tolerance, individual response to surgery, and the extent of the injury can influence the perceived level of pain. Here are some aspects to consider regarding the pain associated with distal biceps repair surgery:

Immediate Postoperative Period: In the immediate postoperative period, patients commonly experience pain and discomfort. Surgical trauma, tissue manipulation, and the effects of anesthesia contribute to this initial pain.

Pain Management Strategies: Surgeons typically prescribe pain medications to help manage postoperative pain. These medications may include analgesics or nonsteroidal anti-inflammatory drugs (NSAIDs). Patients are advised to take these medications as directed.

Individual Variability: Pain experiences are subjective, and individuals may have varying pain thresholds. Some patients may report mild discomfort, while others may describe more significant pain. Effective pain management involves communication with healthcare providers to adjust medications based on individual needs.

Postoperative Swelling: Swelling is a common postoperative response, and it can contribute to pain and discomfort. Elevating the arm and applying ice as directed can help manage swelling and alleviate pain.

Early Mobility and Rehabilitation: Gradual introduction of range of motion exercises and physical therapy is part of the postoperative care. While these exercises are important for recovery, they may initially cause some discomfort.

Pain Duration: The most intense pain is often experienced in the first few days after surgery and tends to decrease as the healing process progresses. Pain levels may vary during different stages of recovery. It's crucial for patients to communicate openly with their healthcare team about their pain levels and any concerns they may have. Healthcare providers can adjust pain management strategies based on individual needs, and patients are encouraged to follow prescribed postoperative care instructions to support a smooth recovery. If you have specific questions or concerns about the expected pain associated with distal biceps repair surgery, it's recommended to discuss them with your orthopedic surgeon. They can provide personalized information based on your unique situation and provide guidance on managing pain during the recovery process.

*JAMA Score: 0*

*Flesch-Kincaid Reading Ease Score: 15.25, Interpretation: Very Difficult*

*Flesch-Kincaid Grade Level: 15.80, Reading level: College*

The JAMA score is 0 out of 4, as the answer does not include any information about authorship, citations/references, disclosures, or dates for when this information was last reviewed or posted. The FRES score is 15.25 as per the formula outlined in the methods section, and as such a ‘Very Difficult’ text [6]. The FKGL score is 15.80 as per the formula outlined in the methods section, and as such a minimum of 16^th^ grade level is required to read the same, which makes this text at a College reading level.

1. How long does a distal bicep repair surgery last?

The duration of distal biceps tendon repair surgery can vary based on several factors, including the surgical technique employed, the complexity of the injury, and individual patient considerations. In general, distal biceps repair surgery is considered a relatively short procedure. Here are some key points to consider regarding the duration of the surgery:

Minimally Invasive Techniques: Many distal biceps tendon repair surgeries are performed using minimally invasive techniques, such as arthroscopy or small incisions. Minimally invasive approaches are associated with shorter surgical times compared to traditional open surgeries.

Surgical Technique: The specific surgical technique chosen by the orthopedic surgeon can influence the duration of the procedure. Some techniques involve reattaching the tendon to its insertion point on the radius bone.

Repair Complexity: The complexity of the distal biceps tendon injury can impact the surgical time. For example, partial tears or less severe injuries may require less time than complete ruptures or cases with associated complications.

Patient Factors: Individual patient factors, such as anatomy, overall health, and any additional medical considerations, can affect the surgical time.

Anesthesia: The type of anesthesia used (general anesthesia or regional anesthesia) can influence the duration of the surgical procedure.

In general, distal biceps tendon repair surgery may take approximately 1 to 2 hours, though this is a rough estimate, and the actual time can vary. Surgeons aim to perform the procedure efficiently while ensuring the accuracy and effectiveness of the repair. It's important for patients to discuss the details of the planned surgery, including the expected duration, with their orthopedic surgeon during preoperative consultations. Surgeons will provide information about the specific surgical approach, anticipated surgical time, and other relevant details to help patients prepare for the procedure and understand what to expect.

*JAMA Score: 0*

*Flesch-Kincaid Reading Ease Score: 12.99, Interpretation: Very Difficult*

*Flesch-Kincaid Grade Level: 16.54, Reading level: College graduate*

The JAMA score is 0 out of 4, as the answer does not include any information about authorship, citations/references, disclosures, or dates for when this information was last reviewed or posted. The FRES score is 12.99 as per the formula outlined in the methods section, and as such a ‘Very Difficult’ text [6]. The FKGL score is 16.54 as per the formula outlined in the methods section, and as such a minimum of 17^th^ grade level is required to read the same, which makes this text at a College graduate reading level.

1. Are distal bicep repair surgery successful?

Distal biceps tendon repair surgery is generally successful in restoring strength, function, and stability to the affected arm when performed under appropriate indications and by experienced surgeons. The success of the surgery depends on several factors, including the nature and extent of the tendon injury, the surgical technique used, the patient's adherence to postoperative care instructions, and individual healing responses. Key factors contributing to the success of distal biceps repair surgery include:

Early Intervention: Timely intervention and surgery soon after the injury, especially for complete ruptures, can contribute to better outcomes. Delayed surgery may lead to tendon retraction and more complex repairs.

Surgical Technique: The choice of surgical technique, whether it be open repair or minimally invasive methods (arthroscopy or small incisions), can impact the success of the surgery. Surgeons tailor the approach based on the specific characteristics of the injury.

Tendon Healing: The ability of the repaired tendon to heal and integrate with the bone is crucial for a successful outcome. The healing process varies among individuals, and factors such as age and overall health can influence tendon healing.

Postoperative Rehabilitation: Adherence to postoperative care, including physical therapy and rehabilitation exercises, is essential for a successful recovery. Physical therapy helps restore range of motion, strength, and functionality in the repaired arm.

Patient Factors: Individual patient factors, such as overall health, lifestyle, and adherence to postoperative instructions, can impact the success of the surgery.

While distal biceps repair surgery is associated with a high rate of success, like any surgical procedure, there can be risks and potential complications. These may include infection, nerve injury, stiffness, or re-rupture of the repaired tendon. Surgeons take steps to minimize these risks, and patients are advised to communicate openly with their healthcare team about any concerns or changes in their recovery. Patients undergoing distal biceps repair surgery should carefully follow their surgeon's postoperative instructions, attend all recommended follow-up appointments, and actively participate in the prescribed rehabilitation program. The majority of individuals experience successful outcomes with improved strength and function following distal biceps tendon repair surgery.

*JAMA Score: 0*

*Flesch-Kincaid Reading Ease Score: 12.78, Interpretation: Very Difficult*

*Flesch-Kincaid Grade Level: 16.78, Reading level: College graduate*

The JAMA score is 0 out of 4, as the answer does not include any information about authorship, citations/references, disclosures, or dates for when this information was last reviewed or posted. The FRES score is 12.78 as per the formula outlined in the methods section, and as such a ‘Very Difficult’ text [6]. The FKGL score is 16.78 as per the formula outlined in the methods section, and as such a minimum of 17^th^ grade level is required to read the same, which makes this text at a College graduate reading level.

The DISCERN scores for all the *Value* questions was 59, which is interpretated as a good source of information. In terms of reliability, questions 22-27 are answered with adequate information provided by ChatGPT, referrers to uncertainty in all the answers, but lacks any information regarding sources, and additional sources of information. The overall quality of information provided about the treatment choices ranges from adequate to excellent, and as such the score for Section 2 and Section 3 of the DISCERN criteria is higher and ranges from 3-5 out of 5.
